# Improvement of the estimation of the infiltration function in surface irrigation systems

**DOI:** 10.1371/journal.pone.0291578

**Published:** 2023-11-16

**Authors:** Mohamed Khaled Salahou, Wei Zhi, Xiaoyuan Chen, Yupeng Zhang, Haishen Lü, Xiyun Jiao

**Affiliations:** 1 School of Biology and Agriculture, Shaoguan University, Shaoguan, China; 2 Northern Guangdong Soil Environment Observation and Research Station, Shaoguan University, Shaoguan, China; 3 Engineering Technology Research Center for Efficient Utilization of Water and Land Resources in North Guangdong, Shaoguan University, Shaoguan, China; 4 State Key Laboratory of Hydrology-Water Resources and Hydraulic Engineering, Hohai University, Nanjing, China; University of Agriculture Faisalabad, PAKISTAN

## Abstract

Surface irrigation systems are widely used on the North China Plain. The design of surface irrigation systems can be improved by developing simulation models including the advanced trajectory, recession trajectory, and infiltration time. Therefore, the objectives of this study were as follows: (1) to evaluate different models to simulate the advanced and recession trajectories, (2) to propose a new method that reduces the required observation data for estimating the infiltration time, and (3) to evaluate the accuracy of the proposed infiltration function based on the modified infiltration time function. Field experiments were conducted. The results indicated that the power function can represent the advanced and recession trajectories well. A modified function that describes the infiltration time has a high correlation and accuracy with the measured data and can be used to estimate the infiltration time. The proposed infiltration function based on the modified infiltration time function is accurate and can be used to estimate the infiltration function.

## 1. Introduction

Evaluating surface irrigation systems mainly depends on the accuracy of estimating the infiltration function [1–6]. The infiltration function can be estimated based on the advance time, recession time, and infiltration time. Water advance time is the relationship between time and distance in waterfront advance, and the water recession time is the relationship between time and distance that begins when the depth of surface water upstream decreases to zero and continues until the surface is drained [7–11]. The infiltration time is the duration it takes for the water to reach a certain depth in the soil.

Several researchers have used the power function to estimate the advanced curve [4, 6–8, 12–16] and the recession curve [7, 8]. The parameters of the power function can be estimated using different methods: in the two-point method, the parameters can be adjusted from observations at two points of the advance/recession curve, [4, 13] best matches the advanced and recession data performed by least squares [12, 16], the regression for the observed advanced and recession data [7, 8], and as a part of a full mathematical model using the numerical solution where the value of the parameters was not stated, but they were estimated at each measuring station as part of the numerical solution [15]. Nie, Li [17] used a second-order equation to estimate the recession time and obtained the equation’s parameters through multipoint regression fitting using observed recession data.

In some studies, the value of the empirical exponent of the power function for the advance curve was determined using furrows of different slopes [18], different types of soils [19], and various other experimental conditions [20–23]. In other studies, the power function’s parameters for advance and recession curves were calibrated based on limited data sets (i.e., one furrow or one border) without identifying the range of these parameters, and the established parameters were used to estimate the infiltration function in the remaining treatments (furrows/borders). However, the variation in these parameters between treatments should be considered and the effect of this variation on the estimated infiltration function should be identified. Furthermore, other functions can also represent the advanced time and recession time, which may be more accurate.

The infiltration time is a critical variable used to estimate the infiltration function. The infiltration time was calculated using simple or complicated mathematical models [24–28] or simply by subtracting the advanced from the recession times [7–9, 17, 29, 30]. In both methods, the accuracy of the infiltration time depends on the accuracy of the estimated advance and recession times and therefore on the measurement of the advance and recession times. Measuring advanced times is easier than measuring recession times, especially in a large-scale project where collecting data requires more field researchers [9]. As a result, the estimated advanced time is more accurate than the estimated recession time. Therefore, reducing the required data and testing other functions to estimate the advanced time and recession time could increase the accuracy of the estimated infiltration function. Hence, the objectives of this study were as follows: (1) to evaluate different mathematical models to estimate the advanced time, recession time, and infiltration time, (2) to improve a mathematical model that increases the accuracy and reduces the required observation data to estimate the infiltration time, and (3) to evaluate the accuracy of the proposed infiltration function based on the modified infiltration time function.

This paper is organized as follows. Section 2 presents different functions to estimate advance, recession, and infiltration times, the suggested method to optimize the empirical parameters of those functions, and the suggested method to estimate the infiltration function. Then, the experimental data used to illustrate and validate the previous methods are described. Section 3 includes the results of the function’s parameters of the advance, recession, and infiltration times and a new method for estimating the infiltration function is proposed. In Section 4, the results of the function’s parameters are provided and the application of the proposed method to estimate the infiltration function is demonstrated. Section 5 summarizes the main conclusions.

## 2. Materials and methods

### 2.1. Advanced time

The water advance curve is formulated as an empirical power equation [8, 14, 17, 19, 31–33] as follows:

$$t_{adv}=ax^{b}$$ (1)

where *t*_*adv*_ is the time of advance (min); x is the distance of advance (m); and a and b are the empirical coefficients, which can be determined for each experiment by minimizing the differences between the observed and estimated (Eq (1)) advance times as follows:

$$\sum_{i=1}^{N}{\left(S_{adv(i)}-O_{adv(i)}\right)}^{2}\to 0$$ (2)

where *S*_*adv*(*i*)_ and *O*_*adv*(*i*)_ are the simulated (Eq (1)) and observed advance time at time i, and N is the number of data pairs.

### 2.2. Recession time

The recession time is approximated by an empirical power equation [7, 8], an exponential equation, or a second-order parabolic equation [31] as follows:

(1) Power equation

$$t_{rec}=T+cx^{d}$$ (3)

(2) Exponent equation

$$t_{rec}=me^{nx}$$ (4)

(3) A second-order parabolic equation

$$t_{rec}=sx^{2}+jx+w$$ (5)

where *t*_*rec*_ is the time of recession (min); x is the distance of advance (m); T is the total time of the advance, recession, and depletion (min) at the head of fields; and c, d, m, n, s, j, and w are the empirical coefficients, which can be determined for each experiment by minimizing the differences between observed and estimated (Eq (3), Eq (4), or Eq (5)) recession times as follows:

$$\sum_{i=1}^{N}{\left(S_{rec(i)}-O_{rec(i)}\right)}^{2}\to 0$$ (6)

where *S*_*rec*(*i*)_ and *O*_*rec*(*i*)_ are the simulated (Eq (3), Eq (4), or Eq (5)) and observed recession time at time i, and N is the number of data pairs.

### 2.3. Infiltration times

The infiltration times (Δ*t*, min) can be obtained based on the differences between advanced and recession times. Hence, the infiltration times can be determined as a function of x by subtracting Eq (1) from Eq (3), Eq (4), or Eq (5). In the Results section, the recession equations (Eq (3), Eq (4), and Eq (5)) are compared, and the equation that gives the best results is used to obtain the infiltration times.

The infiltration times can also be approximated by a second-order parabolic equation as follows:

$$\Delta t=fx^{2}+gx+h$$ (7)

where Δ*t* is the infiltration time (min); x is the distance of advance (m); and f, g, and h are the empirical coefficients, which can be determined for each experiment by minimizing the differences between the observed and estimated values (Eq (7)) infiltration times as follows:

$$\sum_{i=1}^{N}{\left(S_{\Delta t(i)}-O_{\Delta t(i)}\right)}^{2}\to 0$$ (8)

where *S*_Δ*t*(*i*)_ and *O*_Δ*t*(*i*)_ are the simulated (Eq (7)) and observed infiltration time at time i, and N is the number of data pairs.

### 2.4. Infiltration function

The infiltration function is described by the Kostiakov equation, which can be written as follows:

$$Z=k{\Delta t}^{\propto }$$ (9)

where Z is the cumulative infiltration depth, Δ*t* is the opportunity time k, and α are empirical coefficients (k> 0, and 0 ≤ α<1).

The simple postirrigation volume balance (PIVB) [34, 35] is used to solve the k and α parameters based on the following equations [36–38]:

$$k=\frac{V_{z}}{{SUM}_{1}}$$ (10)

$${SUM}_{1}=\sum_{j=2}^{N_{x}}\frac{1}{2}({\Delta t}_{j}^{\propto }+{\Delta t}_{j-1}^{\propto }).(x_{j}-x_{j-1})$$ (11)

Another way to solve the Kostiakov equation using PIVB is by defining the k and α parameters as a function of *t*_100_, the time needed to infiltrate 100 mm [36].


$$\propto =0.675-0.2125{log}_{10}(t_{100})$$ (12)

$$k=\frac{100}{t_{100}^{\propto }}$$ (13)

### 2.5. Border irrigation experiments

In this study, border irrigation experiments were conducted in the town of Nanpi in Hebei Province, China, at a longitude, latitude, and elevation of 116°40’E, 38°06’N and 20 m, respectively. Winter wheat was planted in October 2014 and harvested in the middle of June 2015. The soil texture at the site is classified as silt loam (67.02% silt, 25.19% sand, and 7.79% clay on average), with a mean bulk density of 1.39 g/cm^3^ for the 1 m soil depth.

### 2.6. Infiltration parameters

The Kostiakov equation parameters obtained by the WinSRFR model [10, 11, 39] based on Eq (10), and Eq (11) can simulate the border irrigation process well. The input data included the irrigation requirement, the advanced and recession times at each measurement distance within each border (every 5 m along the border length), the border dimensions (measured using a measuring tape in the field), and the elevations along the border length (measured using an optical level) were recorded for border irrigation. The average reported slope in each block varied from border to border and throughout the irrigation season. Salahou, Jiao [40] published the details of the calculation process for a closed-end border irrigation performance evaluation model considering a distance-based cutoff, and the calculation results are only summarized in this section.

Since the infiltration parameters obtained were well calibrated, the distribution of the infiltration water amount simulated by the WinSRFR model along the border length can be regarded as the measured results, which can be used to test the estimated infiltration water amount distribution function in this study. The details of the layout of the border and measuring points can be found in [40]. The details of the border irrigation are listed in Table 1

**Table 1:** *Inflow rate*, *average slope*, *Manning’s roughness*, *inflow duration*, *advance time*, *CR*, *infiltrated volume*, *and KE equation coefficients for each irrigated border* ^a^.

Block	Border Number	Average slope	Average inflow rate (l/s/m)	Average inflow rate (l/s/m)	Inflow duration (min)	Advance Duration (min)	Cutoff ratio (CR)	Kostiakov coefficients		Manning coefficients	Infiltrated volume (m3)
								a	k mm/hr^a^	n	
A	2	0.0014	6.87	6.89	18.80	27.03	0.8	0.77	143.541	0.09	28.67
	3	0.0022	7.08		17.10	24.27	0.8	0.77	136.168	0.09	26.88
	4	0.0023	6.91		20.70	25.93	0.85	0.77	163.205	0.09	31.75
	5	0.0016	6.85		21.00	26.65	0.85	0.77	134.902	0.09	31.93
	6	0.0015	6.78		22.40	25.63	0.9	0.77	140.11	0.095	33.72
	7	0.0011	6.87		22.80	26.13	0.9	0.77	147.039	0.09	34.77
	8	0.0017	6.84		23.00	26.67	0.9	0.77	166.224	0.09	34.93
B	10	0.0022	4.87	4.94	21.20	28.92	0.8	0.68	104.607	0.095	22.92
	11	0.0023	4.82		24.80	32.7	0.8	0.68	134.966	0.06	26.54
	13	0.0024	5.00		26.20	30.5	0.85	0.68	130.067	0.065	29.08
	14	0.0025	4.93		23.70	29.3	0.85	0.68	122.236	0.06	25.94
	16	0.0014	5.06		24.10	27.58	0.9	0.68	122.771	0.06	27.07
C	19	0.0027	2.78	2.81	42.40	48.23	0.9	0.57	90.835	0.06	26.17
	21	0.0026	2.73		44.70	51.83	0.9	0.57	92.39	0.06	27.09
	22	0.0029	2.84		50.50	52.67	0.95	0.57	99.226	0.06	31.84
	25	0.0029	2.88		51.30	51.33	1	0.57	100.717	0.06	32.80

^a^ Data from the research of Salahou, Jiao and Lü [40]

### 2.7. Statistical analysis of the results

Two statistical indicators were used to analyze the goodness-of-fit between the observations and simulations: (1) the root mean square error (RMSE) and (2) the coefficient of determination, which are calculated as follows:

$$RMSE=\sqrt{\frac{1}{N}\sum_{i=1}^{N}{\left(S_{i}-O_{i}\right)}^{2}}$$ (14)

$$R^{2}=1-\frac{\sum_{i=1}^{N}{\left(S_{i}-O_{i}\right)}^{2}}{\sum_{i=1}^{N}{\left(O_{i}-\bar{O}\right)}^{2}}$$ (15)

where *S*_*i*_ represents the predicted value, *O*_*i*_ represents the observed value, $\bar{O}$ is the mean value of all observed events at time i, and N is the number of data pairs. The RMSE indicates the agreement between the predicted and observed values. R^2^ ranged between 0 and 1.

In addition to the previous statistical indicators, maximum, minimum, mean, standard deviation (*σ*), and coefficient of variation (Cv) [41] were used to describe the estimated parameters. X_m_, *σ*, and Cv can be calculated using the following equations:

$$X_{m}=\frac{1}{N}\sum_{i=1}^{N}x_{i}$$ (16)

$$\sigma ={\left[\sum_{i=1}^{N}\frac{{\left(O_{i}-O_{m}\right)}^{2}}{\left(N-1\right)}\right]}^{\frac{1}{2}}$$ (17)

$$C_{v}=\frac{\sigma }{X_{m}}$$ (18)

where σ is the standard deviation; *xi* is the estimated parameters of the ith point; *X*_*m*_ is the mean value; *N* is the number of data points; and *C*_*v*_ is the coefficient of variation, which reflects the variation in the estimated parameters (i.e., when C_V_ < 0.1 represents weak variability; 0.1 ≤ CV ≤ 1.0 represents moderate variability, and C_V_> 1.0 represents strong variability) [42].

## 3. Results and discussion

### 3.1. Advancement time

**Fig 1:**
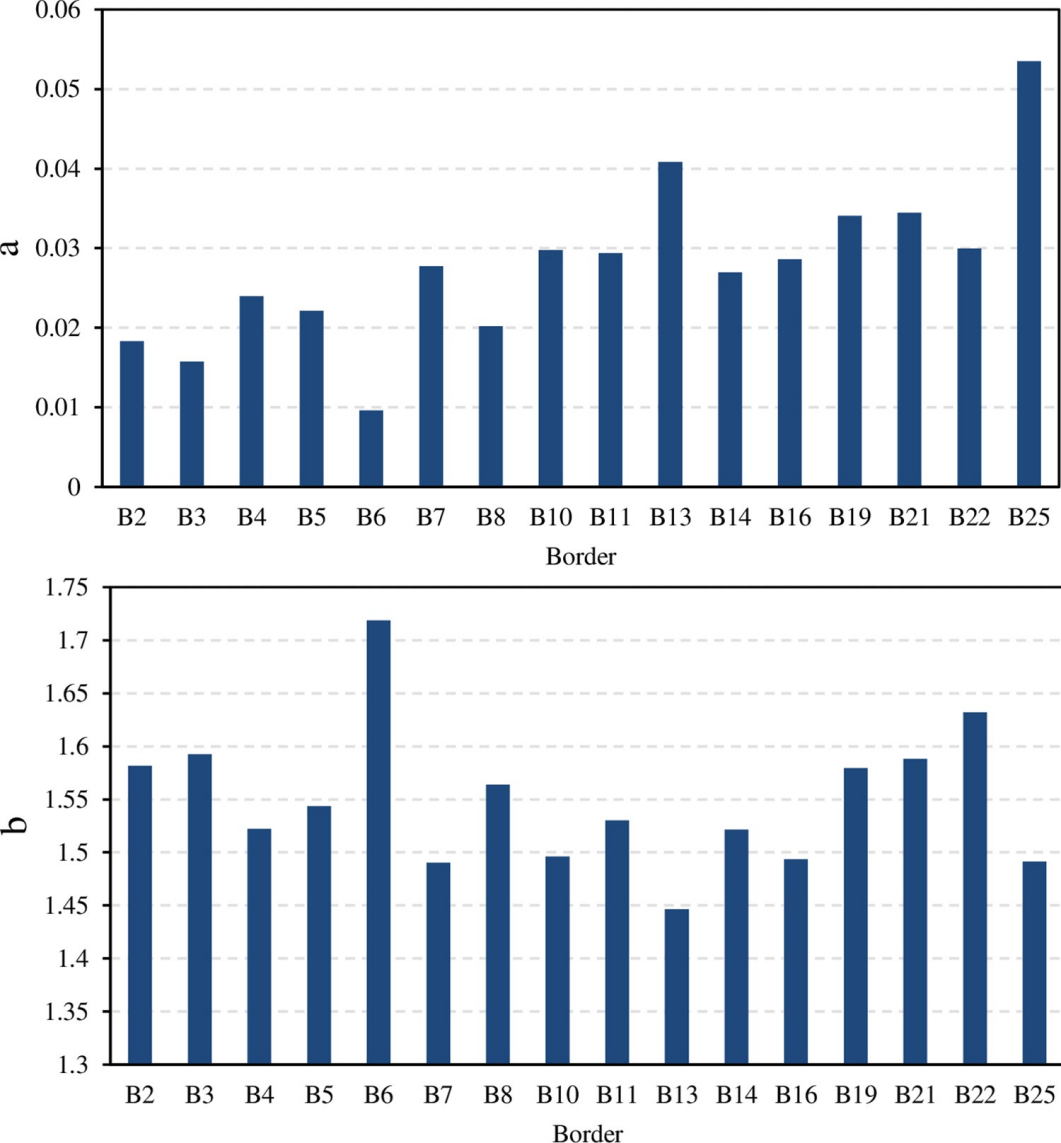
The empirical parameters (a, and b) of the advance equation Eq (1).

**Fig 1** shows the advance equation parameters a and b (Eq (1)).

A statistical analysis was conducted on the parameters of the power function (a, and b) in Table 2. The maximum, minimum, mean, range, standard deviation σ, and coefficient of variation Cv are shown in Table 2. The variation range of coefficient a was 0.0096-0.0535 min·m^-b^, the mean was 0.0278 min·m^-b^, and the coefficient of variation was 0.370, which indicated moderate variability. The variation range of coefficient b was 1.4465 to 1.7186, the mean is 1.5495, and the coefficient of variation was 0.043, indicating relatively weak variability.

**Table 2:** *Statistical characteristics of fitting parameters of the advance equations (Eq (1))*.

Formula	Parameter	Min	Max	Average	range	σ	Cv
Eq (1)	a (min·m^-b^)	0.0096	0.0535	0.0278	0.0439	0.0103	0.370
	b	1.4465	1.7186	1.5495	0.2721	0.0664	0.043

**Fig 2:**
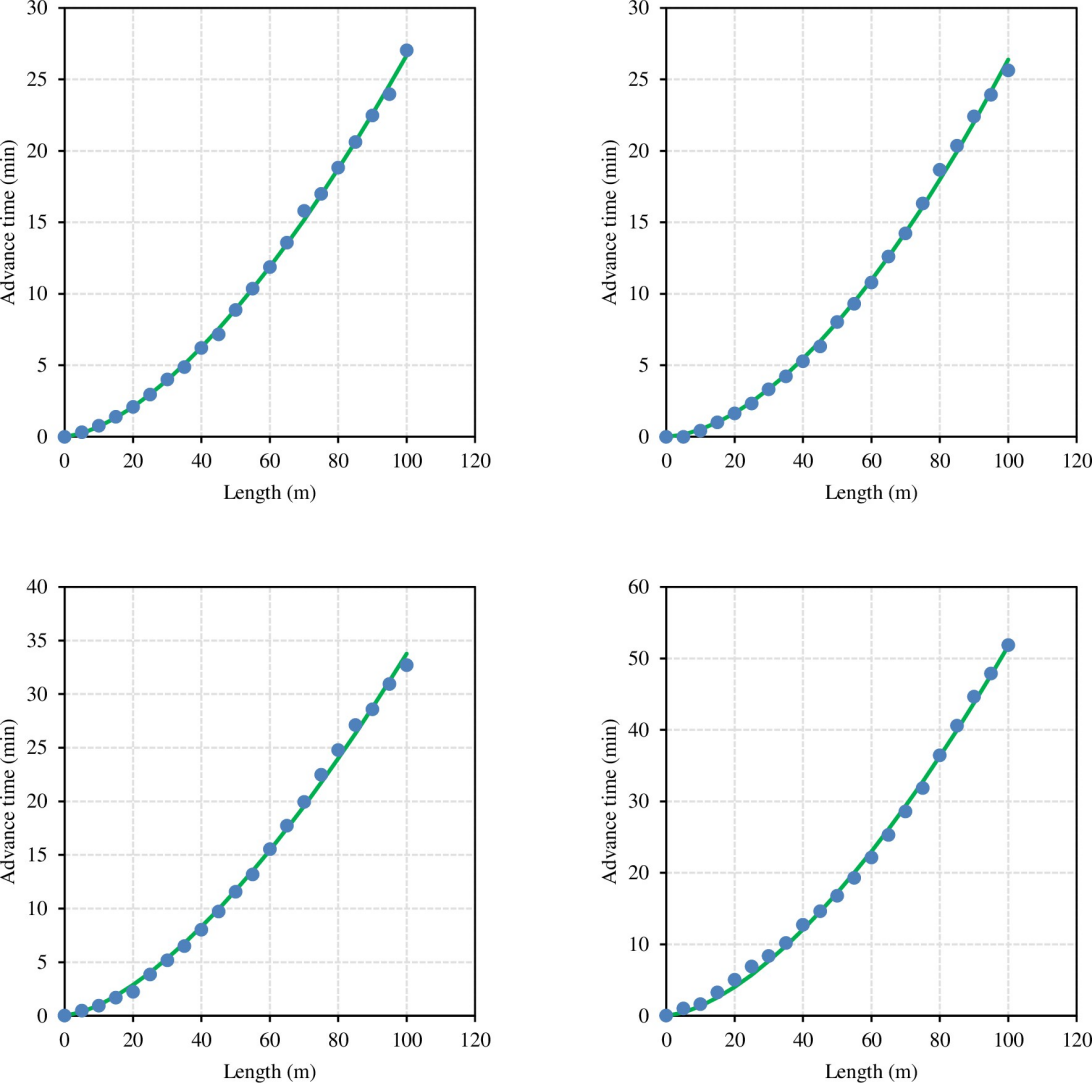
Measured (dots) and simulated (lines) advance curves (Eq (1)) of (a) border 2, (b) border 6, (c) border 11, and (d) border 21.

**Fig 2** shows the advance curve of borders B02, B06, B11, and B21. The fitting curve exhibited a high degree of agreement. Even after the water cut off, the water advancement process was well in line with the power function relationship. The results showed that the advance process conformed to the power function relationship. The R^2^ was between 0.9961 and 0.9997, indicating a very strong correlation. The RMSE did not exceed 0.98 min, and the curve fitting was excellent.

### 3.2. Recession time

**Fig 3:**
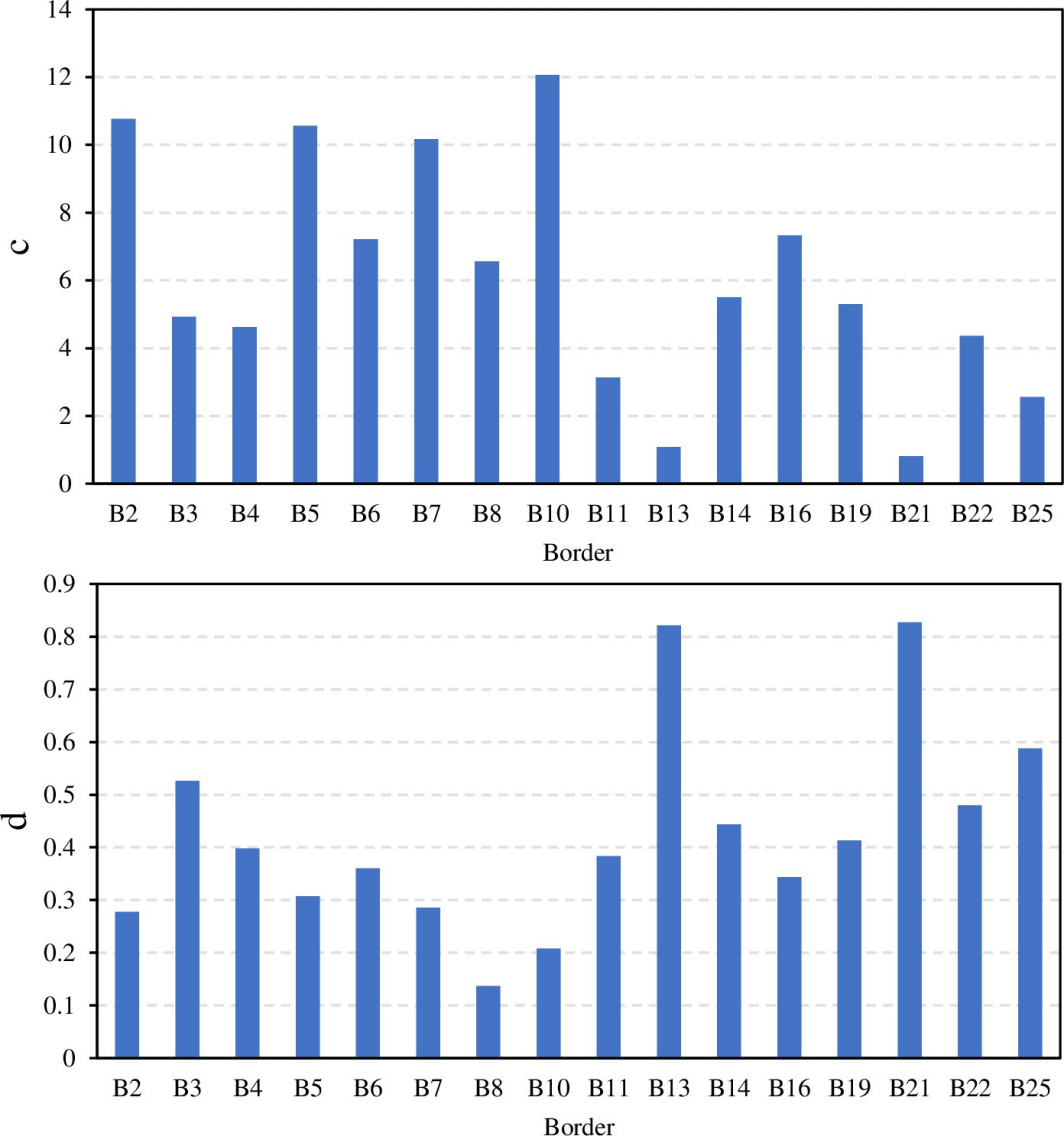
Empirical parameters (c, and d) of the recession equation in Eq (3).

The power function (Eq (3)) was used to fit the surface water flow recession process in border irrigation. **Fig 3** shows the results of the power function’s empirical parameters (c and d). The parameters c and d (Eq (3)) were scattered, and the trend in the change with the influence of the inflow rate was not clear. The d value was less than 1.0, and the recession curve was convex.

**Fig 4:**
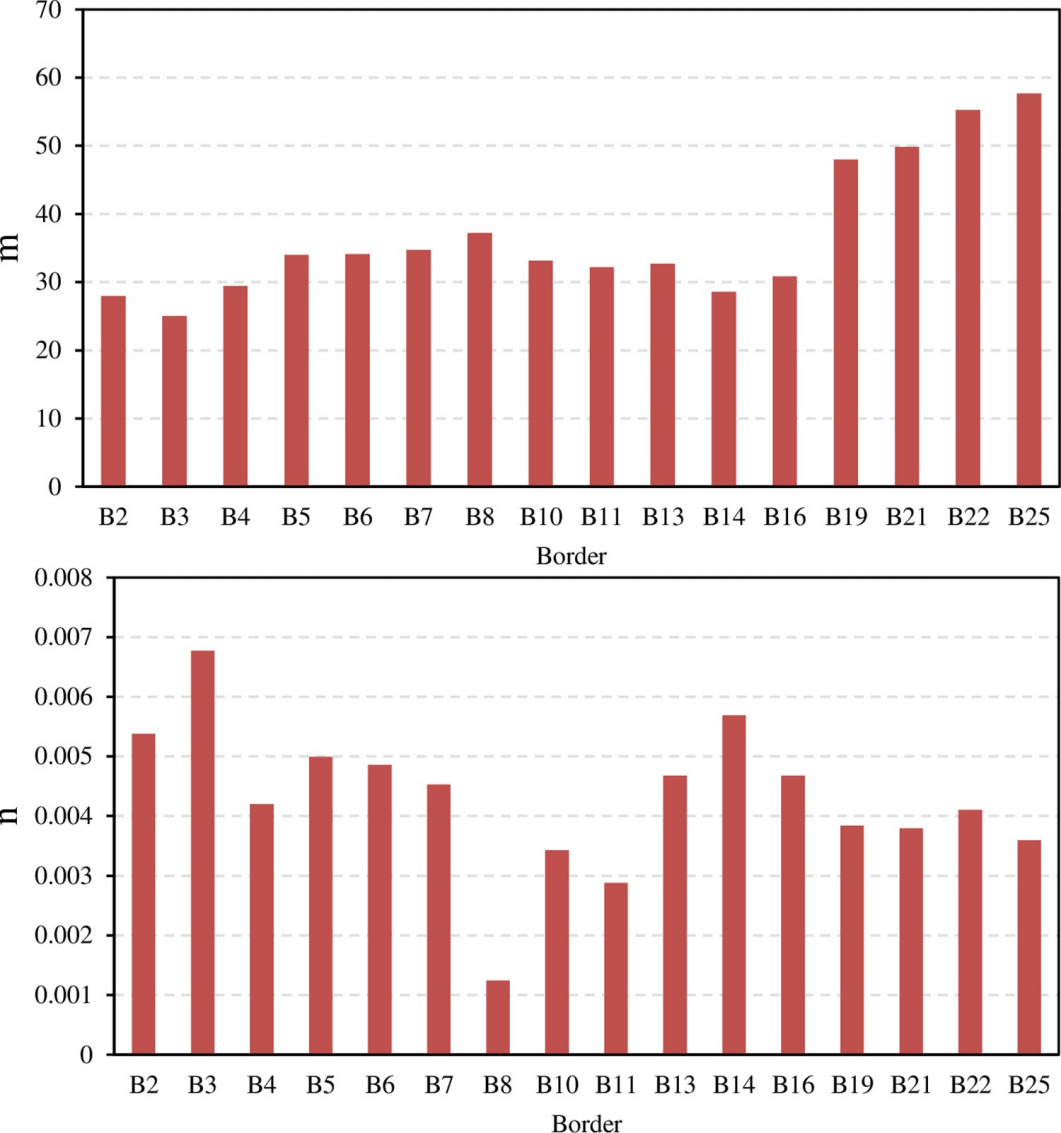
Empirical parameters (m and n) of the recession equation in Eq (4).

The exponential function (Eq (4)) was also used to fit the recession process of the border irrigation events. The variation range of the empirical parameters m and n is small, and both increase with the decrease in inflow rate (**Fig 4**).

**Fig 5:**
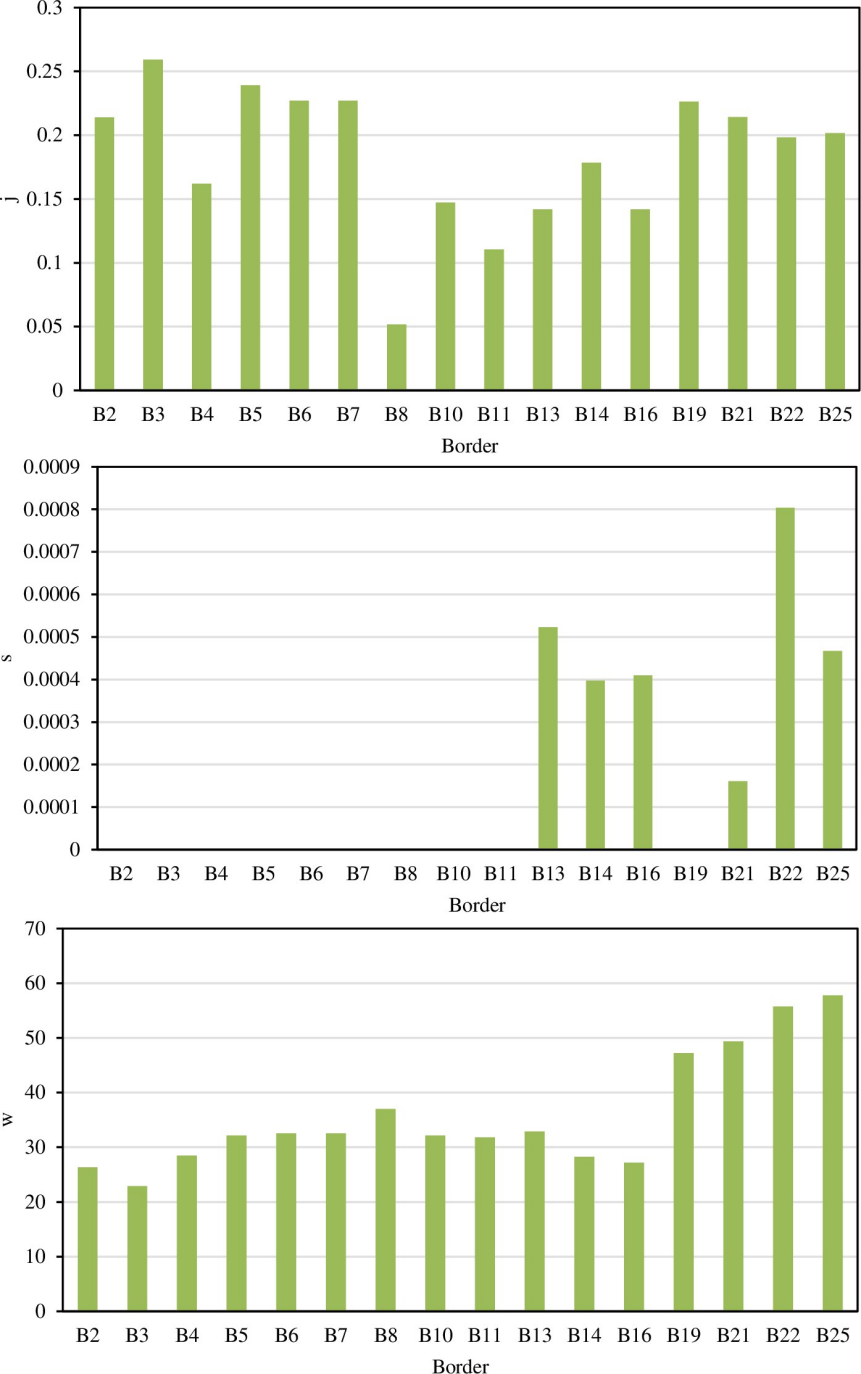
The empirical parameters (s, j, and w) of the recession equation Eq (5)).

The third method for estimating the recession curve involves a second-order parabolic function (Eq (5)). **Fig 5** shows the variation range of the empirical parameters s, j, and w. The parameter s was small and equal to zero at most borders, converting Eq (5) into a straight-line equation. [13] represented the recession curve by a straight-line equation, but they divided the trajectory into two straight lines.

The statistical characteristics of the parameters of the power function (Eq (3)), the exponential function (Eq (4)), and the second-order parabolic function (Eq (5)) are shown in Table 3. Among the parameters, the variation range of the border recession time T was the largest, ranging from 18.83 to 55.50 min, and the second was the coefficient m, which ranged from 25.075 to 57.680 min. The coefficients s and n have the smallest variation range, and the ranges were only 0.001 and 0.006, respectively. In terms of variability, the coefficients of variation of the coefficients c and s were the largest, at 0.569 and 1.505, respectively, indicating strong variability, and the other coefficients all showed moderate variability.

**Table 3:** *Statistical characteristics of fitting parameters for the recession equations (Eq (3), Eq (4), and (Eq (5))*.

Formula	Parameter	Min	Max	Average	range	σ	C_v_
Power Function Eq (3)	T (min)	18.83	55.50	32.01	36.67	11.77	0.37
	c (min·m^-d^)	0.821	12.065	6.066	11.244	3.451	0.569
	d	0.137	0.827	0.425	0.690	0.193	0.453
Exponential function Eq (4)	m (min)	25.08	57.68	36.93	32.61	10.05	0.272
	n (m^-1^)	0.001	0.007	0.004	0.006	0.001	0.291
A second-order parabolic equation Eq (5)	s (min·m^-2^)	0.000	0.001	0.000	0.001	0.000	1.505
	j (min·m^-1^)	0.052	0.259	0.184	0.208	0.055	0.297
	w (min)	22.906	57.766	35.914	34.860	10.669	0.297

**Fig 6:**
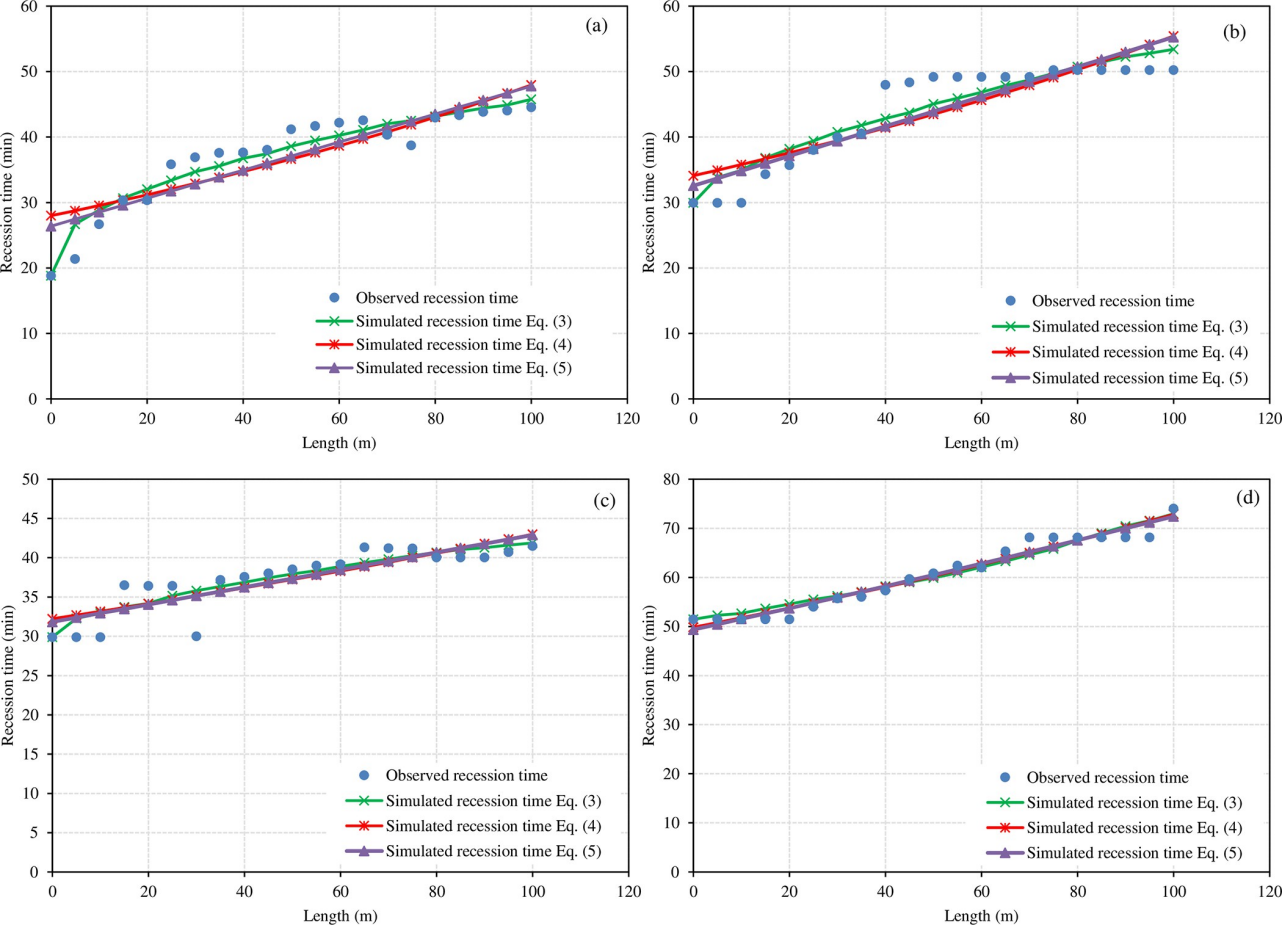
Measured and simulated (Eq (3), Eq. (4), and (Eq (5)) Recession curves of (a) border 2, (b) border 6, (c) border 11, and (d) border 21.

**Fig 6** shows the fitting results of the recession curve of the B02, B06, B11, and B21 borders. The exponential function fit poorly on the gradual recession process of border irrigation, especially at the head of the border. In addition, due to the limitation of the formula itself, the exponential function cannot describe the recession process of the convex shape of some borders well. The R^2^ by the exponential function (Eq (4)) was between 0.147 and 0.956. Except for B07, the R^2^ was low, only 0.147, and the correlation was not significant. The slope of the first part of the B07 border (0-15 m) was steep, while the first half (15-50 m) was low-lying, and the water infiltration time of the field surface here was long, which did not conform to the trend of exponential function change, resulting in a low fitting correlation coefficient. The RMSE was between 1.31 and 5.52 min, which did not differ greatly from the RMSE in the result of the power function (Eq (3)) (2.53 min).

The power function fitting curve did not have a problem describing the shape of the recession curve. The R^2^ by the power function was between 0.605 and 0.9997, showing a very significant correlation. The RMSE was between 0.14 and 4.28 min, which was slightly larger than the fitting result of the advanced process.

The second-order parabolic function (Eq (5)) described the recession curve well. The R^2^ was between 0.155 and 0.0.957, showing a very significant correlation. The RMSE was between 1.31 and 5.33 min, which was similar to that of the exponential function.

Therefore, compared with the previous three methods (Eq (3), Eq (4), and (Eq (5)), the exponential function (Eq (4)) has a simpler form and does not require the determination of the beginning of the recession phase (T). However, the formula form of the exponential function (Eq (4)) determines that the fitting curve of the recession process can only be a downward convex shape, which is inconsistent with the measured data in some fields. The results of 16 borders showed that the power function was better than the other functions. The average RMSE fitted by the power function was 2.53 min lower than that of the other functions (Eq (4), and (Eq (5)), and the average R^2^ was 0.858 higher than that of the other functions.

### 3.3. Infiltration time

Based on the previous results, Eq (3) best represents the recession trajectory. Hence, we determine the infiltration time as a function of x by subtracting Eq (1) from Eq (3) as follows:

$$\Delta t=T+cx_{rec}^{d}-ax^{b}$$ (19)

We noted in the previous sections that b and d are not strong empirical parameters, and the difference between their averages is approximately 0.8, so we can rewrite Eq (19) as follows:

$$\Delta t=T+px^{r}-qx^{r+0.8}$$ (20)

The infiltration time is described by Eq (7), Eq (19), and Eq (20), respectively, and compared with the measured results. The RMSE and R^2^ results are shown in Table 4. Among the above three methods, the RMSE conforms to the following relationship: method 1 > method 2 > method 3, and the correlation coefficient shows the opposite law. The calculation results of Eq (20) have the best correlation and the highest accuracy with the measured data, which can describe the infiltration time curve of border irrigation. This method (Eq (20)) is used to estimate the infiltration function in the following section.

**Table 4:** *The RMSE and R*^*2*^ *of the measured and simulated values (Eq (7), Eq (19), and Eq (20)) of the infiltration times*.

Formula	Index	Min	Max	Average
Eq (7)	RMSE (min)	1.721	4.883	2.956
	R^2^	0.446	0.944	0.776
Eq (19)	RMSE (min)	1.564	17.881	4.338
	R^2^	0.333	0.966	0.773
Eq (20)	RMSE (min)	1.541	4.366	2.408
	R^2^	0.609	0.963	0.843

### 3.4. Infiltration function

The soil infiltration process can be described by the Kostiakov model Eq (9), and the infiltration time at each point in the border field satisfies Eq (20). Hence, the distribution formula of the infiltration water amount along the border length direction can be expressed as follows:

$$Z=K{\left(T+px^{r}-qx^{r+0.8}\right)}^{\propto }$$ (21)

As mentioned in the methodology section, the distribution of the infiltration water amount simulated by the WinSRFR model along the border length can be regarded as the measured results, which can be used to test the estimated infiltration water amount distribution function using Eq (21). The infiltration functions in the 16 borders are described by Eq (21), and the parameters (k, and α) of Eq (21) were calculated based on Eq (13), and Eq (12) (See section 2.4 for obtaining the Kostiakov parameter, and see section 3.3 for fitting coefficient values such as T, p, q, and r)

Table 5 shows the results of the RMSE and R^2^ values of the infiltration function (Eq (21)) of the 16 borders. The mean square error RMSE is between 3.32 and 6.94 min, and the average value of R^2^ is 0.825.

**Table 5:** *The RMSE and R*^*2*^ *of the measured and simulated values (Eq (21)) of the infiltration function*.

Number of Border	B2	B3	B4	B5	B6	B7	B8	B10	B11	B13	B14	B16	B19	B21	B22	B25
RMSE min	3.99	6.49	5.04	5.53	3.85	4.43	6.94	4.69	4.09	3.32	4.20	4.19	3.88	3.34	5.54	4.73
R2	0.888	0.609	0.857	0.818	0.870	0.855	0.896	0.845	0.947	0.854	0.629	0.683	0.880	0.951	0.779	0.829

**Fig 7:**
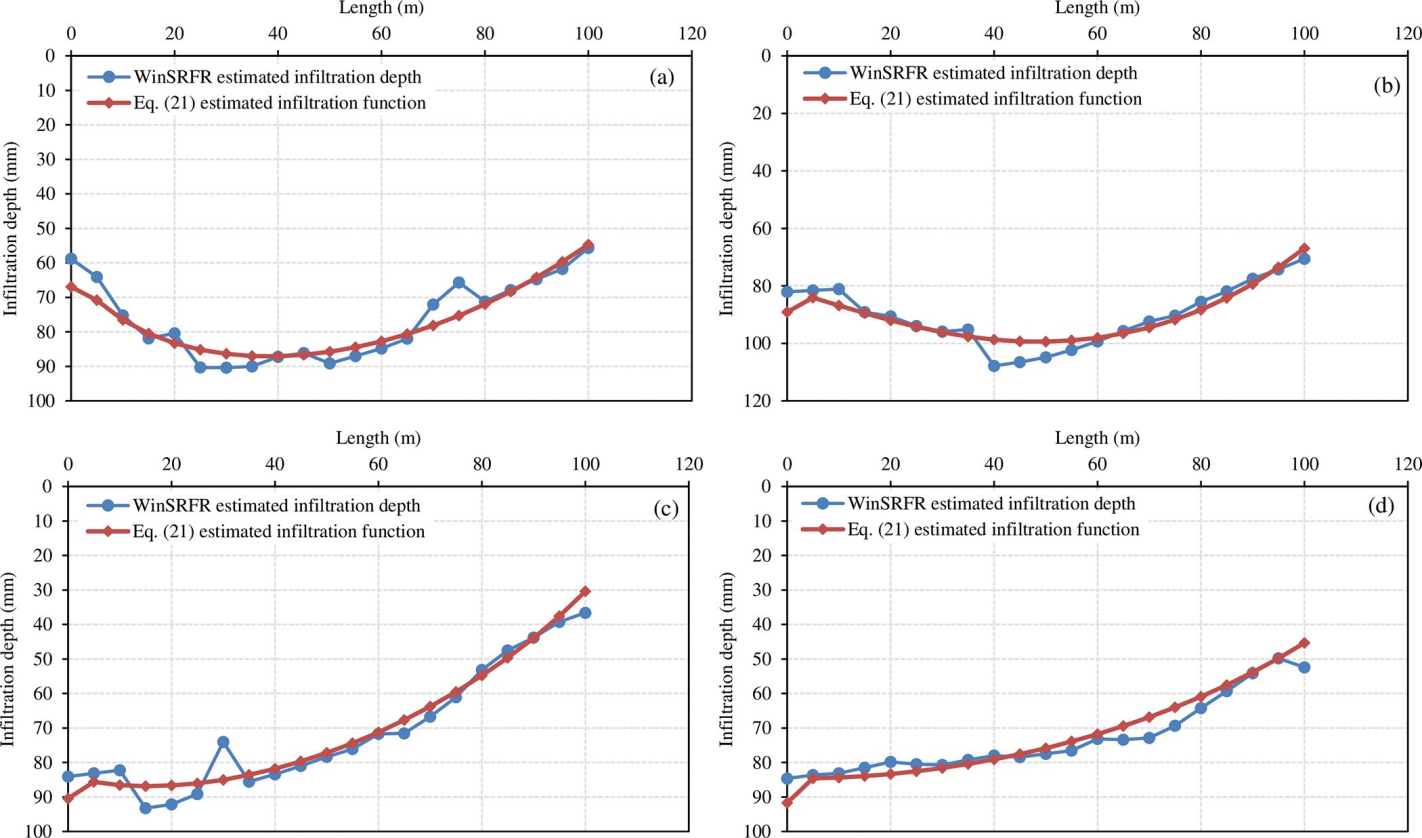
Compression of model results between WinSRFR and Eq (21) for the infiltration function of (a) border 2, (b) border 6, (c) border 11, and (d) border 21.

The infiltration functions of the B02, B06, B11, and B21 borders are shown in **Fig 7**. The calculated value of the infiltration function using Eq (21) is in good agreement with the simulated value using the WinSRFR model with little difference. Hence, we conclude that the infiltration water distribution function established in this work is reasonable, and the calculation results are accurate.

## 4. Discussion

The advance power law with an exponent (b) between 1.44 and 1.72 can represent a different advance rate with time, with an average of 1.55, which is within the basic range of the previous studies. Amer and Amer [7], Amer [8] estimated the advanced power law with an exponent of 1.42. The range of b values reported in other studies under various conditions. For example, Serralheiro [18] reported b values between 0.52 and 0.94 for Mediterranean soil for furrows of different slopes. Alvarez [19] estimated b values between 0.58 and 0.72 for different types of soil for furrow irrigation systems. In another study, Khatri and Smith [20] reported b values for 27 furrows with an average of 0.86, and Ebrahimian and Liaghat [22] reported b values for 5 furrows and 6 borders with averages of 0.63 and 0.58, respectively. Therefore, the b value is a function of several factors, such as soil moisture, soil type, surface slope, and irrigation system. Hence, fitting power functions to field data increases the accuracy of the estimated infiltration function.

Relationships for estimating recession times in blocked-end borders have been previously proposed [35, 43], but they can only be used with prior knowledge of infiltration. This process consists of two distinct phases. In the upper part of the field, water will be lost primarily through free drainage, and recession will occur relatively quickly. For the lower portion of the field, water can only be lost by infiltration. Hence, the recession will take much longer and will be approximately linear with time if the slope is uniform. In the real world, recession times will depart from linearity due to the nonuniformity of the bottom slope. **Fig 6** presents comparisons of measured and simulated recession time values based on the different fitting procedures tested. The measured data are somewhat atypical of blocked-end borders, in which some ponding occurs at the end of the field. In these tests, the inflow was cut off and carefully timed to prevent ponding [40]. A fundamental problem with the PIVB method is that one equation is available to solve for all unknown parameters of the infiltration equation being used. Even when dealing with the two parameters of the Kostiakov equation, an infinite number of solutions can be proposed. To overcome this problem, an empirical relationship developed by Merriam and Keller [34] was used. In this study, the developed empirical relationship for recession times is applicable only to the conditions of the study. To apply this method under other conditions, recession times must be measured, and a new empirical relationship must be developed for those new conditions. Thus, the problem of needing to measure recession times in the field was partially solved. The recession time can be measured in some experiments, and then the method presented in this study can be applied to the remaining experiments. Although the recession time measurements are inaccurate in comparison in advance, which, in turn, justifies the proposal to use estimates instead of measured values, the advance time measurements are subject to considerable uncertainty just as recession ones, even though those measurements are more precise. The arrival of the irrigation stream to a measuring station is affected by other factors besides infiltration, e.g., hydraulic resistance, bottom slope undulations, soil clods, vegetation, cracking, etc. In a border, the stream typically does not advance uniformly across the width. Thus, advanced measurements may be precise, but they may not accurately represent infiltration. While recession time measurements are imprecise, they provide more information about long-term infiltration rates than the advance times. The former only provides information about the early part of the infiltration process. Recession times can be difficult to obtain manually, but with current developments in electronics, we can expect to develop sensors that not only make it easier and less costly to collect recession data but also measure flow depths during the postadvance phase of irrigation. Those depths are critical to improving the accuracy of infiltration estimates and to determining hydraulic resistance independently from infiltration. However, sensors are not available at many research sites.

## 5. Conclusion

Establishing the infiltration function in a surface irrigation system is critical. Different methods were presented to estimate the advanced trajectory, recession trajectory, infiltration time, and infiltration functions. The results showed that the advanced trajectory can be represented well with the power function Eq (1). By comparing the three methods for estimating the recession trajectory, the power equation Eq (3) best fit the observation data. When the total time of the advance, recession, and depletion at the head of borders was not collected, the second-order parabolic equation Eq (5) can be an alternative to estimate the recession trajectory. The results showed that the s parameter of the second-order parabolic equation was small and equal to zero at most borders, converting Eq (5) into a straight-line equation. The modified function (Eq (20)), which describes the infiltration time of border irrigation, had the best correlation and the highest accuracy with the measured data and, therefore, can be used to estimate the infiltration time. A new method was improved to estimate the water distribution along the field. The results show that the new infiltration function (Eq (21)) is in good agreement with the simulation results obtained from the WinSRFR model. Hence, the infiltration water distribution function established in this work is reasonable, and the calculation results are accurate. The border irrigation system was tested using the established equations in this study. However, a furrow irrigation system could also be tested.
